# Supplementary material for: Redβ177 annealase structure reveals details of oligomerization and λ Red-mediated homologous DNA recombination
Source: Nat Commun. 2022 Sep 26;13:5649. doi: 10.1038/s41467-022-33090-6 (PMC9512822; doi:10.1038/s41467-022-33090-6)
Supplement: Supplementary file 1 — Supplementary Information [file 41467_2022_33090_MOESM1_ESM.pdf]

Supplementary Information for

Redβ_{177} annealase structure shows how it anneals DNA strands during single-strand annealing homologous DNA recombination

Timothy P. Newing, Jodi L. Brewster, Lucy J. Fitschen, James C. Bouwer, Nikolas P. Johnston, Haibo Yu, Gökhan Tolun

Gökhan Tolun's Email: gokhan_tolun@uow.edu.au

This document includes:

Supplementary Figure captions for S1 to S7
All Supplementary Figures
Supplementary Table 1
SI References

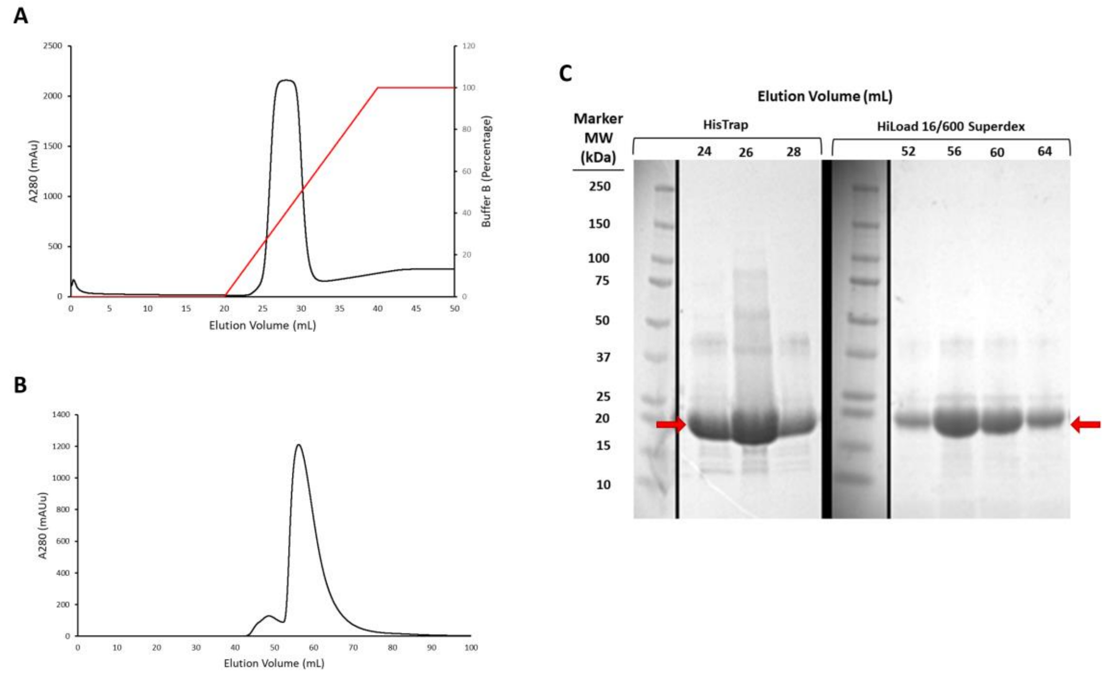

Supplementary Fig. 1. The two-step purification of Redβ$_{177}$. (A) Column profile for immobilized metal ion affinity chromatography of Redβ$_{177}$. (B) Column profile for size exclusion chromatography purification through a HiLoad 16/600 Superdex column. The black line represents the UV (280 nm) absorbance, while the red line represents the increasing the concentration (%) from 10 mM to 500 mM imidazole. (C) SDS-PAGE gel showing bands at the expected size of Redβ$_{177}$, highlighted by red arrows. Gels were not repeated since the cryo-EM imaging also confirmed the bands being Redβ$_{177}$.

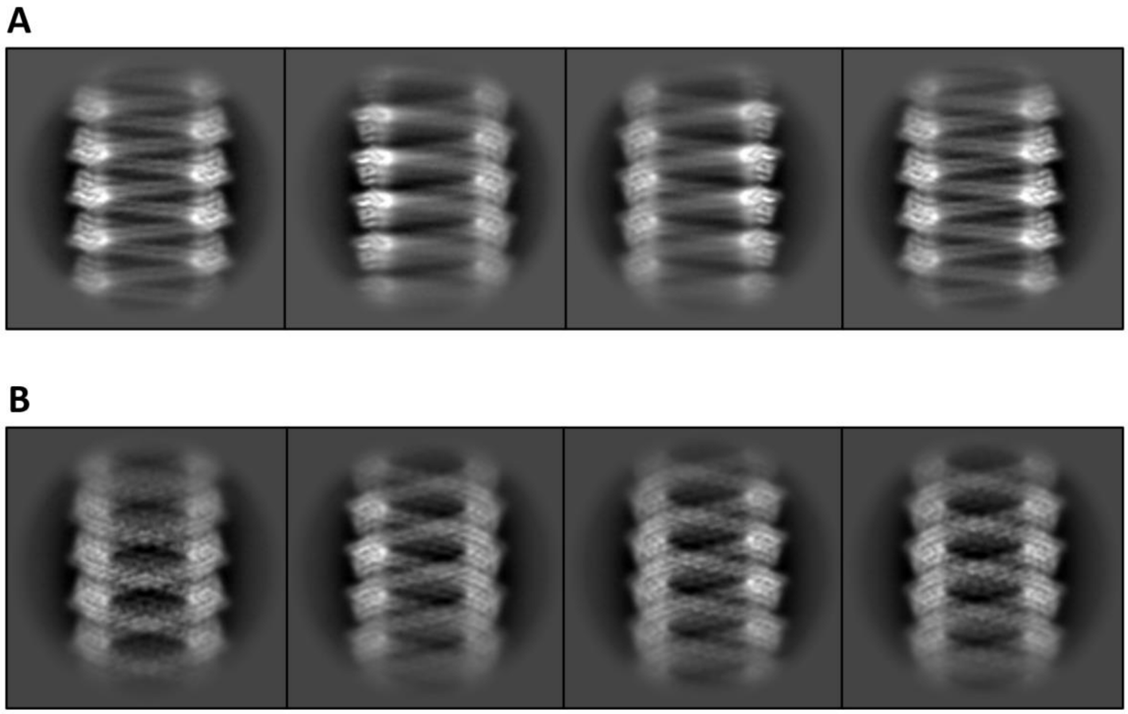

Supplementary Fig. 2. 2D class averages for the 1-start and 2-start helical assemblies. Selected 2D class averages of the Redβ$_{177}$ helical filaments obtained after processing in cryoSPARC (*1*). These include populations of both the 1-start helix (A) and the 2-start helix (B).

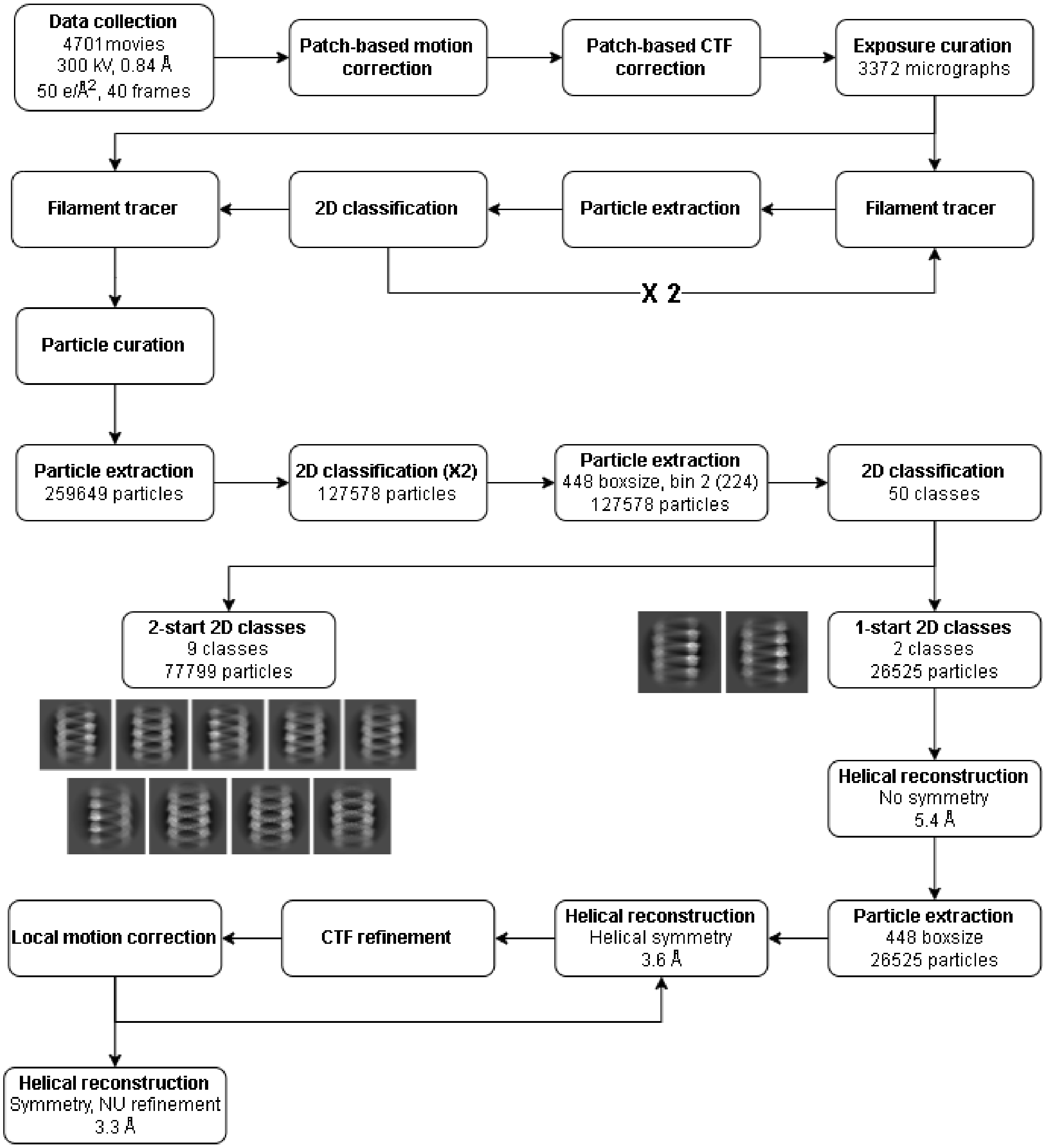

Supplementary Fig. 3. Overall workflow of the cryo-EM data processing. Flowchart shows the cryo-EM data analysis performed, from the initial micrographs to the final reconstruction.

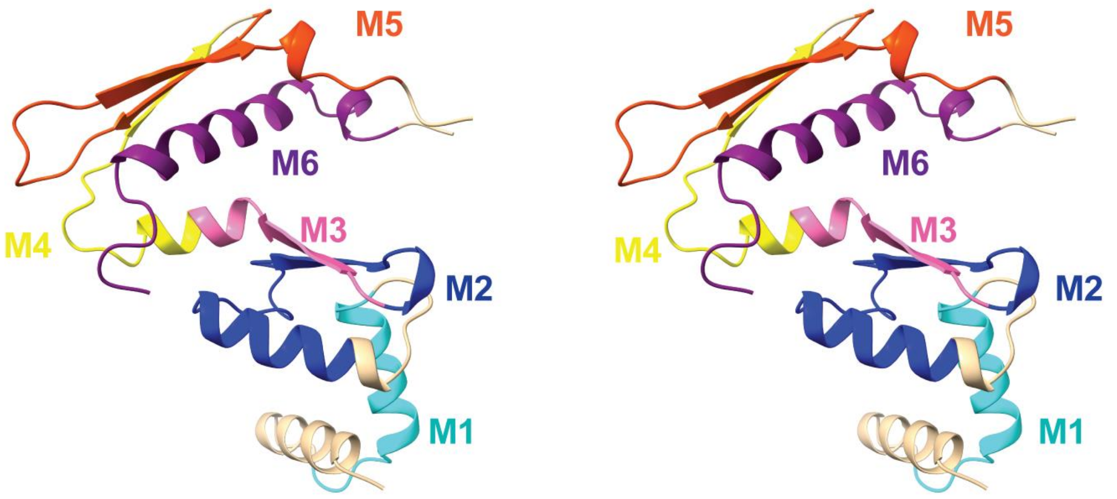

Supplementary Fig. 4. Cross-eye stereo of the conserved motifs from the MSA mapped onto the structure of Redβ$_{177}$. Colors indicate different conserved motifs identified by the MSA (Supplementary Data 1), with 6 out of the 8 motifs being represented in separate colors and numbered. Beige indicates the areas outside of the identified motifs.

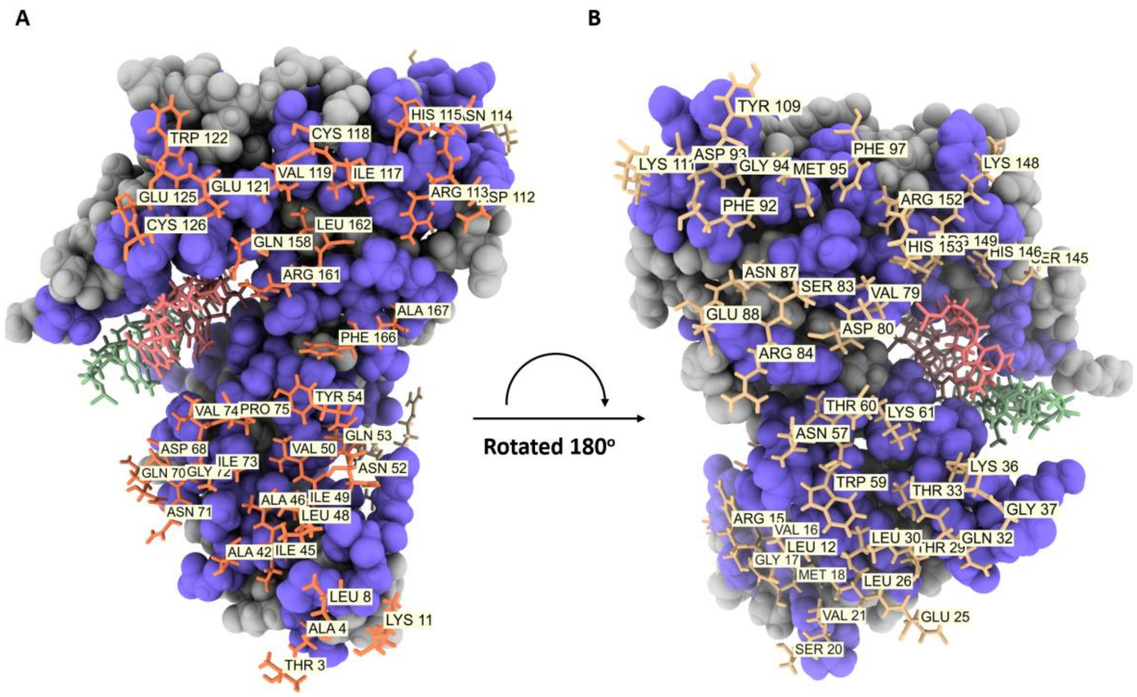

Supplementary Fig. 5. Redβ$_{177}$ Interacting residues. Residues involved in side-by-side interactions between two Redβ$_{177}$ monomers. (A) and (B) show the two sides of the Redβ$_{177}$ monomer that would be facing each other in the helical assembly.

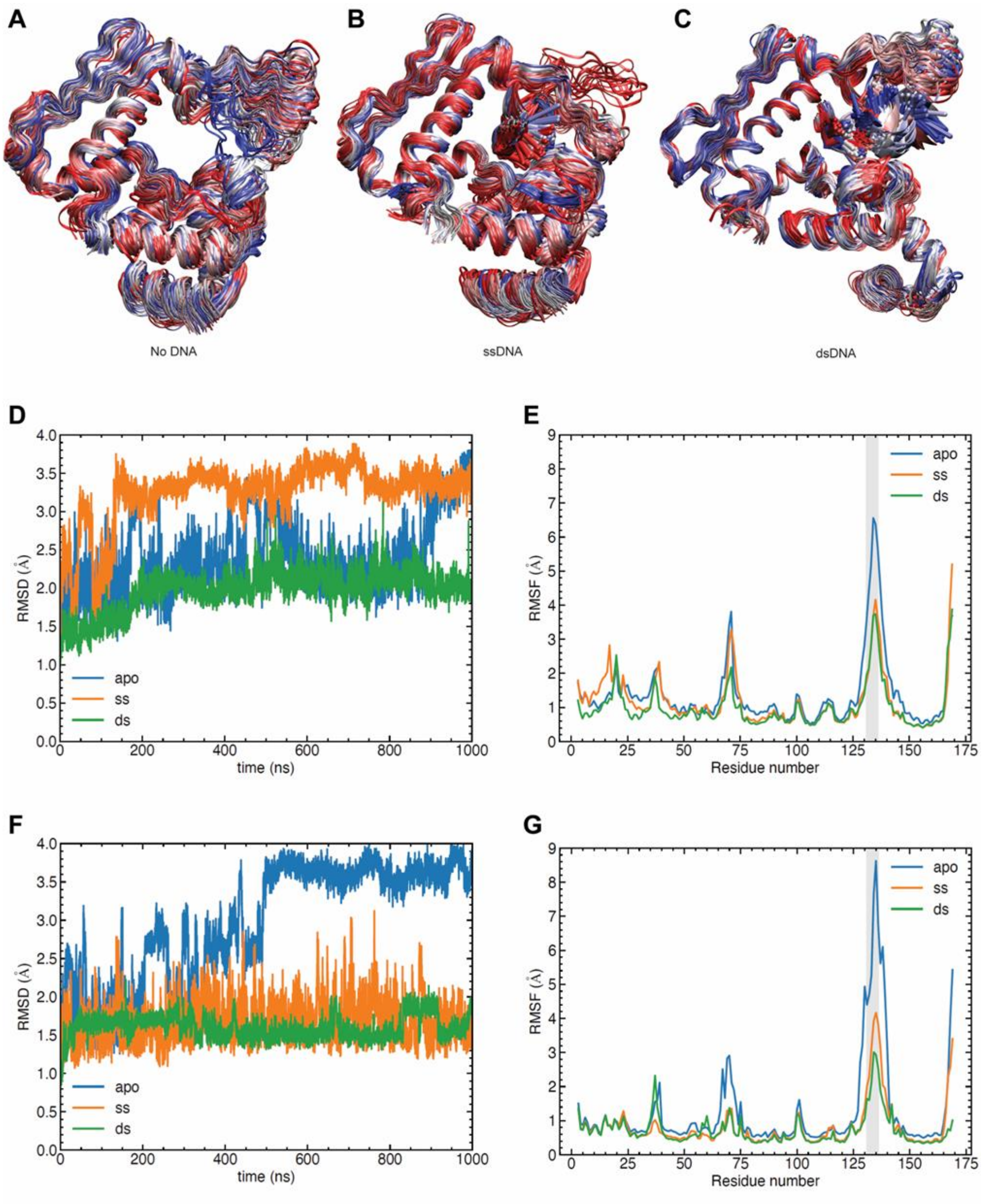

Supplementary Fig. 6. Molecular Dynamics without restraints snapshots. The sampled ensemble of apo (A), ssDNA-bound (B) and dsDNA-bound (C) Redβ$_{177}$ states in the MD simulations of the trimeric systems without any restraints. 100 snapshots with a time interval of 10 ns from 1 µs simulations are shown. The structures were fitted to the backbone atoms for the monomer in the middle of the trimeric system. Blue, silver and red colors correspond to early, mid and late time intervals, respectively. The backbone positional root-mean-square deviations for the middle protein chain from the initial structure as a function of simulation time (D) without any restraints and (F) with a weak harmonic restraints on the two outside monomer and DNA backbone atoms). The Cα atom positional root-mean-square fluctuations for the middle protein chain (E) without any restraints and (G) with a weak harmonic restraints.

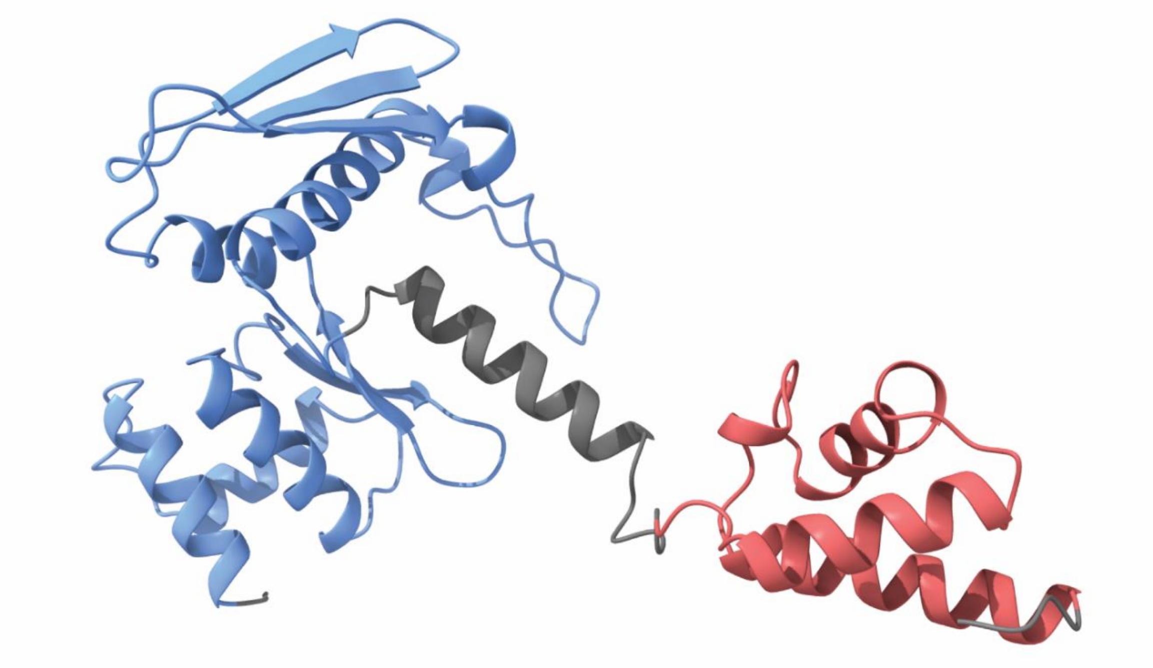

Supplementary Fig. 7. The full-length Redβ structure predicted by AlphaFold (*2*). Blue shows the N-terminal domain that corresponds to the $Red\beta_{177}$ structure in this study, red shows the residues that correspond to the C-terminal domain of Redβ determined by Caldwell *et al.* (*3*), and grey are the residues whose structures were not experimentally determined.

Supplementary Table 1. Cryo-EM data collection, refinement and validation statistics.

	Redβ177-dsDNA (PDB: 7UJL, EMD-26566)
Data collection and processing	
Molecular weight (kDa/nm)	114.18
Magnification (x)	59500
Voltage (kV)	300
Electron exposure (e-/Å2)	50.0
Defocus range (Å)	500-2500
Pixel size (Å/pixel)	0.84
Initial particle images	259,649
Final particle images	26,525
Axial symmetry imposed	C1
Helical rise (Å)	2.078
Helical twist (°)	-12.947
Map resolution (Å)	3.3
FSC threshold	0.143
Map resolution range (Å)	2.8-4.8
Refinement	
Initial model used	N/A
Model resolution (Å)	3.2
FSC threshold	0.5
Model composition	
Non-hydrogen atoms	1447
Protein residues	161
Nucleic acid residues	8
B factors	
Protein	30.00
Nucleic	9.27
r.m.s deviations	
Bond lengths (Å)	0.015
Bond angles (°)	2.075
Validation	
MolProbity score	0.85
Clashscore	0.00
Poor rotamers (%)	0.00
Ramachandran plot	
Favoured (%)	94.90
Allowed (%)	5.10
Outliers (%)	0.00

Supplementary Information References

1. A. Punjani, J. L. Rubinstein, D. J. Fleet, M. A. Brubaker, cryoSPARC: algorithms for rapid unsupervised cryo-EM structure determination. *Nat Methods* **14**, 290-296 (2017).
2. J. Jumper *et al.*, Highly accurate protein structure prediction with AlphaFold. *Nature* **596**, 583-589 (2021).

3. B. J. Caldwell *et al.*, Crystal structure of the Redbeta C-terminal domain in complex with lambda Exonuclease reveals an unexpected homology with lambda Orf and an interaction with Escherichia coli single stranded DNA binding protein. *Nucleic Acids Res* **47**, 1950-1963 (2019).
4. A. M. Waterhouse, J. B. Procter, D. M. Martin, M. Clamp, G. J. Barton, Jalview Version 2--a multiple sequence alignment editor and analysis workbench. *Bioinformatics* **25**, 1189-1191 (2009).

Supplementary information – Original (uncropped) gel images

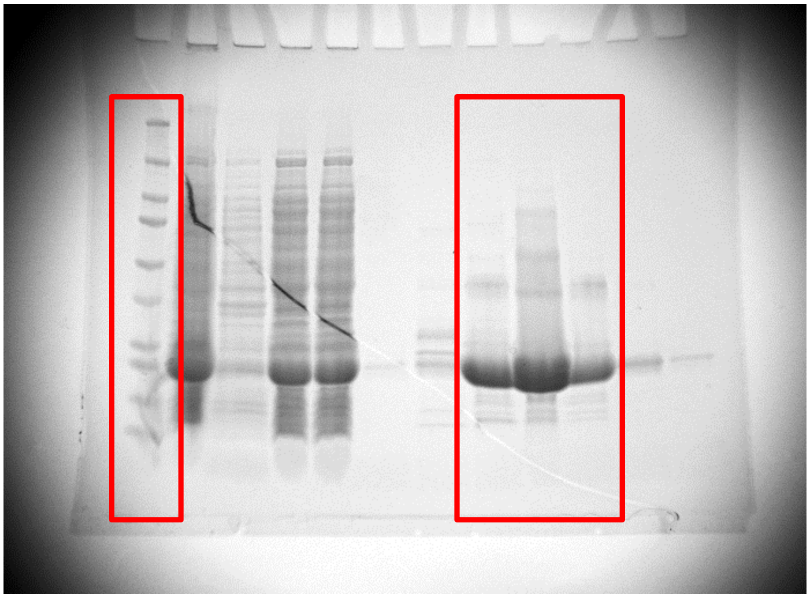

Uncropped version of the left side of the gel figure showing the HisTrap column fractions in Supplementary figure 1.C

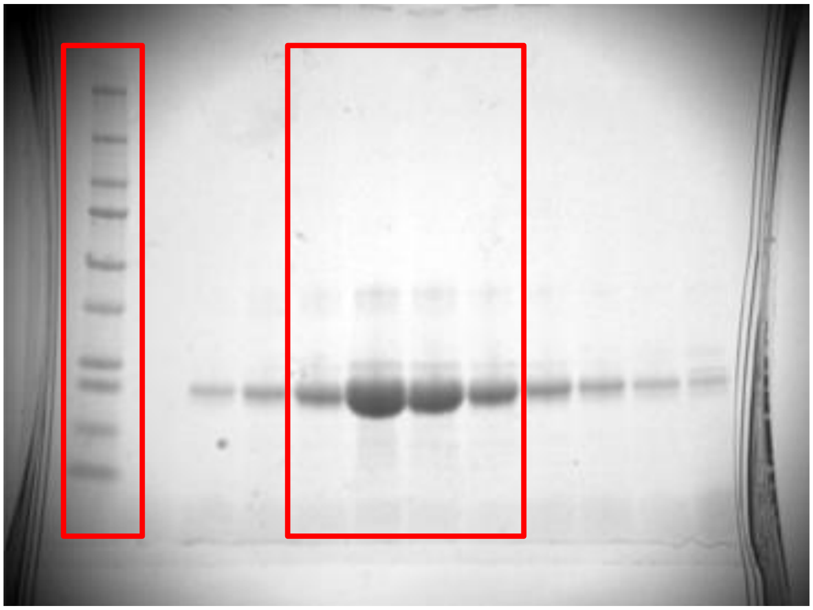

Uncropped version of the right side of the gel figure showing the HiLoad 16/600 Superdex column fractions in Supplementary figure 1.C
