# Supplementary material for: Redβ177 annealase structure reveals details of oligomerization and λ Red-mediated homologous DNA recombination
Source: Nat Commun. 2022 Sep 26;13:5649. doi: 10.1038/s41467-022-33090-6 (PMC9512822; doi:10.1038/s41467-022-33090-6)
Supplement: Supplementary file 2 — Description of Additional Supplementary Files [file 41467_2022_33090_MOESM2_ESM.pdf]

File name: Supplementary Data 1

Description: Multiple Amino Acid Sequence Alignment. The alignment is composed of the top 1000 UniProt Ref90 clusters related to the full-length Redβ amino acid sequence (UniProtKB: P03698). Conserved sequence motifs, based on 50% consensus, are shown (top) with individual residues colored by amino acid side chain property: hydrophobic = orange, negative charge = red, polar uncharged = pink, positive charge = blue and other properties/ambiguous amino acid codes = black. Positions where an amino acid property is conserved, rather than an individual residue, are indicated: hydrophobic = 'h', polar uncharged = 'p', positive charge = '+'. The eight conserved motifs (M1-M8) identified are indicated (top). The alignment is colored following the 'ClustalX' scheme as implemented in Jalview v.2.11.1.4 (4).
