# Supplementary material for: Redβ177 annealase structure reveals details of oligomerization and λ Red-mediated homologous DNA recombination
Source: Nat Commun. 2022 Sep 26;13:5649. doi: 10.1038/s41467-022-33090-6 (PMC9512822; doi:10.1038/s41467-022-33090-6)
Supplement: Supplementary file 3 — Supplementary Data 1 [file 41467_2022_33090_MOESM3_ESM.pdf]

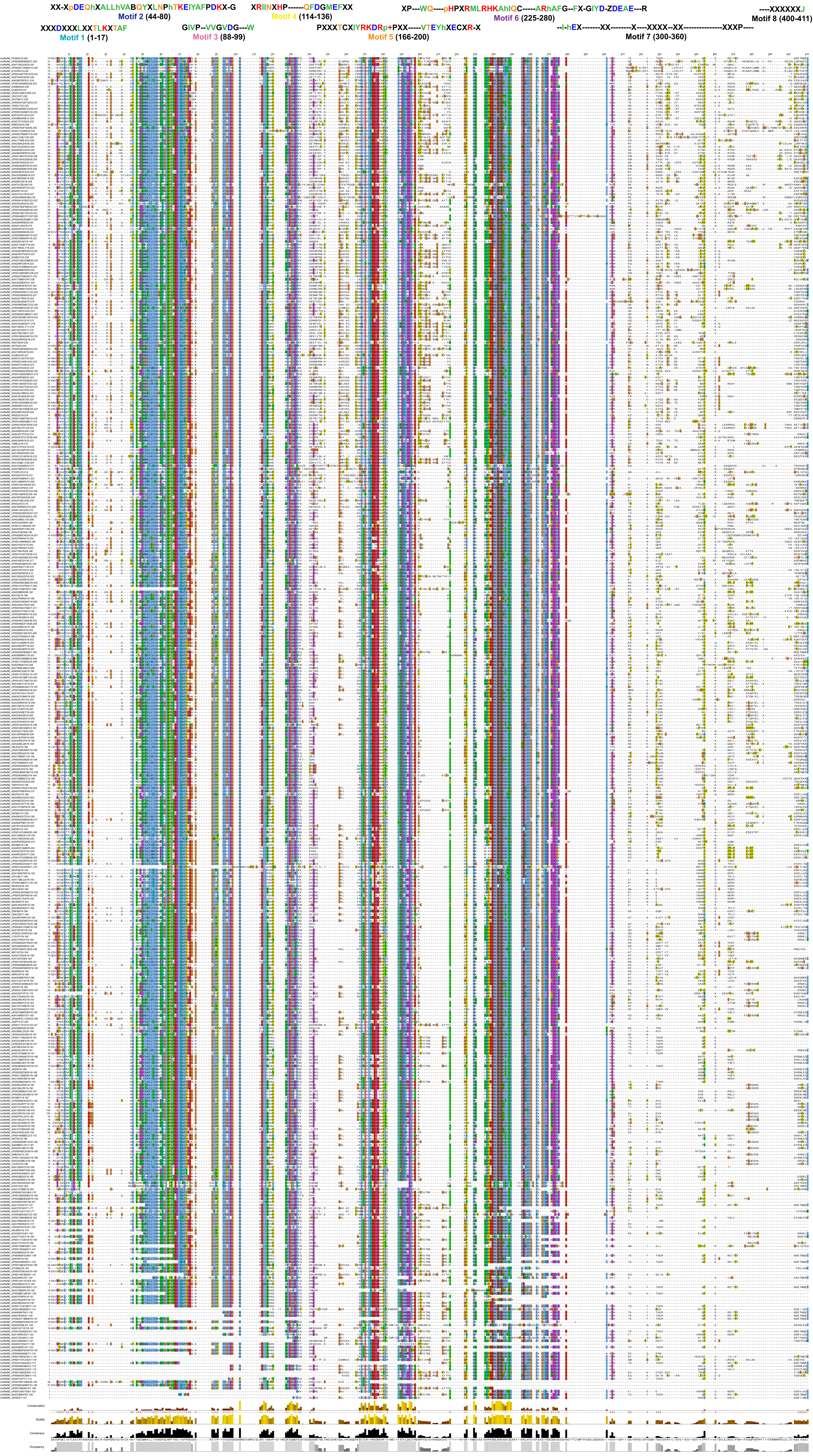

XX-XpDEQhXALLhVABQYXLNPhTKEIYAFPDKX-G
Motif 2 (44-80)
XRIINXHP------QFDGMEFXX
Motif 4 (114-136)
XP---WQ----pHPXRMLRHKAhIQC-----ARhAFG--FX-GIYD-ZDEAE---R
Motif 6 (225-280)
----XXXXXXJ
Motif 8 (400-411)
XXXDXXXLXXTLKXTAF
Motif 1 (1-17)
GIVP--VVGVDG---W
Motif 3 (88-99)
PXXXTCXIYRKDRp+PXX-----VTEYhXECXR-X
Motif 5 (166-200)
--I-hEX-------XX--------X----XXXX--XX-------------XXXP----
Motif 7 (300-360)
Conservation
Quality
Consensus
Occupancy
