# Supplementary material for: Redβ177 annealase structure reveals details of oligomerization and λ Red-mediated homologous DNA recombination
Source: Nat Commun. 2022 Sep 26;13:5649. doi: 10.1038/s41467-022-33090-6 (PMC9512822; doi:10.1038/s41467-022-33090-6)
Supplement: Supplementary file 4 — Reporting Summary [file 41467_2022_33090_MOESM4_ESM.pdf]

nature portfolio

Corresponding author(s): Dr. Gökhan Tolun

Last updated by author(s): May 11, 2022

Reporting Summary

Nature Portfolio wishes to improve the reproducibility of the work that we publish. This form provides structure for consistency and transparency in reporting. For further information on Nature Portfolio policies, see our Editorial Policies and the Editorial Policy Checklist.

Statistics

For all statistical analyses, confirm that the following items are present in the figure legend, table legend, main text, or Methods section.

n/a	Confirmed	
☐	☒	The exact sample size (*n*) for each experimental group/condition, given as a discrete number and unit of measurement
☐	☒	A statement on whether measurements were taken from distinct samples or whether the same sample was measured repeatedly
☒	☐	The statistical test(s) used AND whether they are one- or two-sided<br>*Only common tests should be described solely by name; describe more complex techniques in the Methods section.*
☒	☐	A description of all covariates tested
☒	☐	A description of any assumptions or corrections, such as tests of normality and adjustment for multiple comparisons
☒	☐	A full description of the statistical parameters including central tendency (e.g. means) or other basic estimates (e.g. regression coefficient) AND variation (e.g. standard deviation) or associated estimates of uncertainty (e.g. confidence intervals)
☒	☐	For null hypothesis testing, the test statistic (e.g. *F*, *t*, *r*) with confidence intervals, effect sizes, degrees of freedom and *P* value noted<br>*Give P values as exact values whenever suitable.*
☒	☐	For Bayesian analysis, information on the choice of priors and Markov chain Monte Carlo settings
☒	☐	For hierarchical and complex designs, identification of the appropriate level for tests and full reporting of outcomes
☒	☐	Estimates of effect sizes (e.g. Cohen's *d*, Pearson's *r*), indicating how they were calculated

Our web collection on statistics for biologists contains articles on many of the points above.

Software and code

Policy information about availability of computer code

Data collection	EPU, v2.14.0.3653REL, Thermo Fisher Scientific Inc.<br>Titan TEM User Interface, v 3.10.1, build 54632, Thermo Fisher Scientific Inc.<br>DigitalMicrograph, v3.44.3448.0, Gatan Inc.<br>NAMD (Version 3.0, Open Source, https://www.ks.uiuc.edu/Research/namd/)<br>LocalColabFold (Version 1.0.0, Open Source, https://github.com/YoshitakaMo/localcolabfold)
Data analysis	Cryosparc v3.3<br>ChimeraX v1.3<br>Fiji (Based on ImageJ v1.53)<br>WinCoot 0.9.2<br>ISOLDE 1.3<br>Phenix 1.19.2<br>MolProbity v4.2<br>VMD (Verions 1.9.4a55, Open Source, https://www.ks.uiuc.edu/Research/vmd/)<br>LOOS (Version 3.3, Open Source, https://github.com/GrossfieldLab/loos)<br>MDAnalysis (Version 2.0.0, Open Source, https://www.mdanalysis.org/)<br>MAFFT (Multiple Alignment using Fast Fourier Transform) v.7.480 (freeware)<br>Jalview v.2.11.1.4 (freeware)<br>Geneious Prime v.2021.1.1 (Commercial)

For manuscripts utilizing custom algorithms or software that are central to the research but not yet described in published literature, software must be made available to editors and reviewers. We strongly encourage code deposition in a community repository (e.g. GitHub). See the Nature Portfolio guidelines for submitting code & software for further information.

Data

Policy information about availability of data

All manuscripts must include a data availability statement. This statement should provide the following information, where applicable:

- Accession codes, unique identifiers, or web links for publicly available datasets
- A description of any restrictions on data availability
- For clinical datasets or third party data, please ensure that the statement adheres to our policy

The reconstructed EM density map (EMD-26566) and the atomic model (7UJL) have been deposited to EMDB and PDB databases, respectively, both of which will be released publicly upon publication. A data availability statement has been added to the manuscript.

Field-specific reporting

Please select the one below that is the best fit for your research. If you are not sure, read the appropriate sections before making your selection.

☒ Life sciences ☐ Behavioural & social sciences ☐ Ecological, evolutionary & environmental sciences

For a reference copy of the document with all sections, see nature.com/documents/nr-reporting-summary-flat.pdf

Life sciences study design

All studies must disclose on these points even when the disclosure is negative.

Sample size	Information on sample size is presented in Supplementary Figure 3. No sample size calculations were performed. The number of EM micrographs in the dataset was determined by the maximum volume of data collection possible on the available hardware within the constraints of time allotted to the project, and since the processing of the collected data set resulted in a high-resolution structure sufficient for atomic modeling, no further data collection was needed. The number of helical filaments identified for particle extraction in each micrograph was determined by the concentration of the sample, the random distribution of filament density across the grid, and the subsequently randomly determined local macromolecular crowding in each image. The number of individual particles extracted from the filaments was determined by the sinuosity of the filaments, and the inter-box distance of 55 pixels, which was calculated using the software recommended value of 20% of the estimated helical diameter (55 pixels x 0.84 Å/pix = 46.2 Å from an estimated helical diameter of 230 Å).
Data exclusions	Structure determination by cryo-EM routinely involves iterative data processing involving a number of steps, such as the (2D and 3D) classification of the particles to separate the high quality ones suitable for use in reconstructions from poor ones. Poor quality particles may be due to the inherent heterogeneity in the biological sample prepared and used, or may be caused during the preparation of the sample for cryo-EM imaging. The main processing steps involving data exclusion are summarized briefly in Figure S3, in which the number of particles used and eliminated at each step can be seen.
Replication	During cryo-EM image data processing, data is randomly divided into two halves, and a structure is determined from each one. Then, these two structures are compared to each other for determining the resolution of the reconstructed map. This is referred to as the "gold standard" resolution determination method in the cryo-EM field, which is a built-in assessment of reproducibility.
Randomization	The cryo-EM image data has been collected using an automation software EPU. After the thin-enough for imaging areas in the cryo-EM grids have been manually selected at low magnification at which the particles cannot be seen, high-magnification images used during data processing were acquired automatically by EPU. Hence, introduction of user-bias was minimized during data collection. Moreover, the 2D class averages presented in Figure 1 and Figure S2, and the reconstruction of an anisotropy-free (or low-enough anisotropy) high-resolution structure that allowed atomic modeling from these particles indicates that the orientations of the helical filaments, and therefore, the orientations of the particles generated from them, were randomly distributed in ice. During cryo-EM single-particle analysis, all particles were randomly assigned into classes by the classification algorithm in the first iteration of every classification. For resolution determination via the gold standard FSC method, the particles were randomly assigned to one of two halves and reconstructed independently in order to control for the effects of overfitting. No other randomization is required/relevant in this study.
Blinding	Blinding was not applicable during this study as cryo-EM analysis does not involve allocation of data into experimental groups that are compared with each other.

Reporting for specific materials, systems and methods

We require information from authors about some types of materials, experimental systems and methods used in many studies. Here, indicate whether each material, system or method listed is relevant to your study. If you are not sure if a list item applies to your research, read the appropriate section before selecting a response.

Materials & experimental systems

n/a	Involved in the study
☒	☐ Antibodies
☒	☐ Eukaryotic cell lines
☒	☐ Palaeontology and archaeology
☒	☐ Animals and other organisms
☒	☐ Human research participants
☒	☐ Clinical data
☒	☐ Dual use research of concern

Methods

n/a	Involved in the study
☒	☐ ChIP-seq
☒	☐ Flow cytometry
☒	☐ MRI-based neuroimaging
